# An algorithm to improve the accuracy of emergency weight estimation in obese children

**DOI:** 10.11604/pamj.2018.31.90.13821

**Published:** 2018-10-05

**Authors:** Mike Wells, Lara Nicole Goldstein

**Affiliations:** 1 Division of Emergency Medicine, Faculty of Health Sciences, University of the Witwatersrand, Johannesburg, South Africa

**Keywords:** Body weight, resuscitation, pediatric obesity

## Abstract

**Introduction:**

During medical emergencies in children, accurate and appropriate weight estimations may ultimately influence the outcome by facilitating the delivery of safe and effective doses of medications. Children at the extremes of habitus, especially obese children, are more at risk of an inaccurate weight estimation and therefore may be more at risk of medication errors. The objective was therefore to develop an algorithm to guide accurate emergency weight estimation in obese children.

**Methods:**

Relevant medical evidence was reviewed regarding weight estimation and its role and timing in the resuscitation of obese children. This was used as the basis for a weight-estimation algorithm.

**Results:**

There was limited evidence regarding the way the weight-estimation systems should be used in obese children other than that the dual length- and habitus-based systems were the most accurate. The methods included in the algorithm were the Broselow tape, the Mercy method, parental estimates, the paediatric advanced weight prediction in the emergency room/ eXtra Length-eXtra Large (PAWPER XL) tape and the Traub-Johnson formula. The algorithm recognised several ways in which weight estimation could be tailored to the clinical scenario to estimate both ideal and total body weight.

**Conclusion:**

Weight-estimation in obese children must be conducted appropriately to avoid medication errors. This algorithm provides a framework to achieve this.

## Introduction

The process of weight estimation during the management of paediatric emergencies is very important. Failure to obtain an early and accurate weight estimate may be a major impediment to resuscitative care for two reasons: firstly, the process of obtaining a weight estimation may cause uncertainty and delay which will prevent the timely administration of necessary medications as well as other weight-dependent interventions (e.g. defibrillation); secondly, inaccurate weight estimation may result in dosage errors, with the resultant potential for patient harm [1]. Failure to obtain a timeous, accurate and appropriate weight estimation must be considered a medical error [2]. Ultimately, the accuracy of the weight estimation may determine the efficacy and safety of resuscitative drug treatment and failure to achieve accurate dosing may lead to increased mortality, especially in obese or underweight children who are at highest risk of inaccurate weight estimations [3]. There is a wealth of evidence to support the use of the newer dual length- and habitus-based weight-estimation systems when weight cannot be measured in an emergency. The most-studied examples of these systems are the Mercy method and the PAWPER (or PAWPER XL) tape. The older, one dimensional age- or length-based methodologies have been superseded, primarily because of their inaccuracy but also because of the cognitive burden they can impose on the treating healthcare professional [4]. Not only is the accuracy of weight estimation important, but the *method* as well as the ease with which it is obtained is crucial.

With the increasing prevalence of obesity in low-, middle- and high-income countries, healthcare providers may well be faced with children of vastly different body habitus and body composition in the resuscitation room. The use of weight estimation methods that estimate only total body weight (TBW) may not be sufficient for all children, since obese children require an estimation of ideal body weight (IBW) in addition, for certain drug dose calculations [5]. The use of IBW alone would also not be appropriate as TBW is still required for many dose calculations in obese children. For normal weight and underweight children TBW is used for all drug dose calculations. In underweight children IBW might far exceed TBW, which could result in a dangerous overdose of potentially harmful medications [6].

How the estimation of TBW and IBW should be accomplished during emergency care in obese children, in a manner that is accurate, safe and easy-to-perform, has not previously been considered. There is limited evidence on the harmful effects of dosage errors resulting from poor weight estimation during resuscitative care, but it cannot be considered to be good medical practice to tolerate significant medication errors resulting from inaccurate or inappropriate weight estimations [7]. It would be useful, therefore, to establish guidelines on how to approach weight estimation at the time of initiation of emergency medical care in obese children. The aim of this study was to create an evidence-based algorithm for weight estimation, to be employed during the management of medical emergencies in children, with a focus on how weight estimation should be achieved in obese children. The constraints were that the accuracy, ease-of-use and time-to-completion of weight estimation had to be balanced against the potential for aggravating the cognitive load, which could impact negatively on resuscitative care.

## Methods

### Search strategy

An online search using Scopus, MEDLINE, Google Scholar and Google was conducted using the following search terms: “paediatric weight estimation”, “weight estimation children”, “Broselow tape”, “Mercy method” and “resuscitation aid”. The articles were screened to retrieve relevant information on the accuracy of weight estimation systems, the performance of weight estimation systems in obese and underweight children specifically, the requirements for training with existing weight-estimation systems and the performance of weight estimation systems under stressful conditions.

### Creation of the algorithm

The algorithm was created with the emergency interventions that might be required for three common emergency scenarios in mind: a child in cardiac arrest, a child that required urgent airway management and a child with convulsive status epilepticus. The algorithm was also developed with the objective of optimising weight estimation for all children, including obese and underweight children, to facilitate safe and accurate drug dose determination.

## Results

There was very little evidence on any aspect of weight estimation other than accuracy. A summary of the evidence is shown in ****Table 1****.

**Table 1:** Summary of the evidence evaluated: the direct and indirect evidence for each topic is displayed; there is very little evidence on any aspect of weight estimation other than accuracy, much of which is of low grade

	Healthcare provider Guess	Parental Estimate	Age-based formulas	Broselow Tape	Mercy Method	PAWPER XL Tape
Accuracy of weight estimation systems for estimating TBW	Very inaccurate - should not be used	Accurate if parent has a recent weight (especially if child weighed in their presence)	Very inaccurate - should not be used	Inconsistent across populations, has low-intermediate accuracy and probably should not be used	Very accurate across a wide range of populations; not evaluated in very obese populations	Very accurate across a wide range of populations, moderately accurate in severely obese children
Performance of weight-estimation systems in underweight populations	No evidence	Limited evidence; similar results to normal weight populations	Overestimate weight significantly	Overestimates weight substantially	Very accurate	Very accurate
Performance of weight-estimation systems in obese populations – estimation of TBW	No evidence	Not accurate in overweight and obese children (indirect evidence)	Underestimate weight significantly	Underestimates TBW substantially	Accurate except in severely obese patients	Moderately accurate, less accurate than the Mercy method
Performance of weight-estimation systems in obese populations – estimation of IBW	No evidence	No evidence	The European Paediatric Life Support formula predicts IBW with moderate accuracy	The tape can provide an accurate estimate of IBW in obese children	Cannot estimate IBW	Estimates IBW very accurately, simultaneously with TBW
Training requirements for weight-estimation systems	No evidence	No evidence	Easily forgotten	Very high incidence of errors in simulation studies	Higher errors with less experienced raters	Decreased accuracy if habitus scoring performed poorly
Performance of weight-estimation systems under stressful conditions	No evidence, but unlikely to be better than study conditions	Unknown, but of concern	Calculation errors higher under stressful conditions	Unknown, errors more likely than during simulation studies	Unknown	Unknown
Weight estimation systems and integration with resuscitation aids	No integration	No integration	No integration	Has only been studied with the use of supplementary reference materials	No designated integration	Designed to be used with colour-coded materials; linked to the Flipper card, EDDC book and/or EDD4Children app
Cognitive burden of weight estimation systems	No evidence	No evidence	Calculation errors are common in all levels of healthcare providers – may make age-formulas unreliable	Negligible burden for weight-estimation; supplementary material required for drug dosing information	Calculation errors are common in all levels of healthcare providers – may make Mercy method unreliable	Negligible burden; supplementary material required for drug dosing information

### Summary of findings used to create the algorithm

Information on weight estimation in obese children was very sparse with only low-grade evidence available. The relevant evidence could be divided into two main groups of findings, as summarised below.

### What are the most appropriate dose scalars for obese children?

Several recent reviews have provided some guidance about appropriate dosing of emergency medications in obese children. Although there is some difference between anaesthetic and emergency medicine dosing, the principles of which weight descriptors should be used have been well elucidated [5, 8, 9]. In obese children, lipophilic drugs–which account for about half of the drugs that are commonly-used during paediatric emergencies–should generally be dosed using TBW as a scalar or weight descriptor. Hydrophilic drugs should be dosed to IBW in obese children to avoid overdosing if dosed to TBW [5]. Some drugs, such as phenytoin, require an intermediate dosing scalar to achieve optimum dosing. This adjusted body weight (ABW) is generally calculated from TBW, IBW and a defined constant.

### Which weight estimation systems are the most accurate in obese children?

**Age-based formulas:** The evidence was very clear that age-based formula estimations of TBW are very inaccurate in all children–even more so in obese children–and should not be used. Formulas are also hard to remember and even the simplest calculations are vulnerable to errors during emergencies [10]. There is some preliminary evidence that some age-based formulas, especially the European Paediatric Life Support formula may be useful to estimate IBW [11].

**Length-based formulas:** The Traub-Johnson and Traub-Kichen formulas have not been shown to estimate TBW with acceptable accurately, but can predict IBW with a high degree of accuracy [12].

**Broselow tape:** The tape has been shown to be inaccurate in both underweight and obese populations, with potentially deleterious effects on patient safety [13, 14]. The tape does predict IBW with a great deal of accuracy, however [15].

**Guesses and estimations:** The use of healthcare providers’ guesses of TBW to calculate drug doses have been shown to be grossly inaccurate and potentially harmful to children [7, 16]. There is no evidence on the ability of healthcare providers to estimate IBW. Parental estimates of TBW can be very accurate [13]. Parental estimates may not be as accurate in overweight and obese children as in normal weight children, however, unless the parent can recall the result of a recently scale-measured weight [17].

**Dual length- and habitus-based systems:** From the evidence, it appears that accurate TBW estimation can be best achieved using two-dimensional, dual length- and habitus-based systems such as the Mercy method and the PAWPER or PAWPER XL tape, especially in obese and underweight children. These systems are far more accurate than other, older systems [18]. Accurate IBW estimation can be achieved using the Broselow tape, the PAWPER or PAWPER XL tape and the length-based formulas (such as the Traub-Johnson formula) [19].

### Algorithm

**Figure 1:**
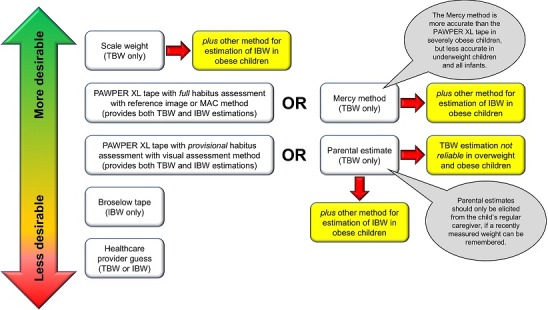
A hierarchy of weight estimation systems ranked according to level of accuracy and ease-of-use

**Figure 2:**
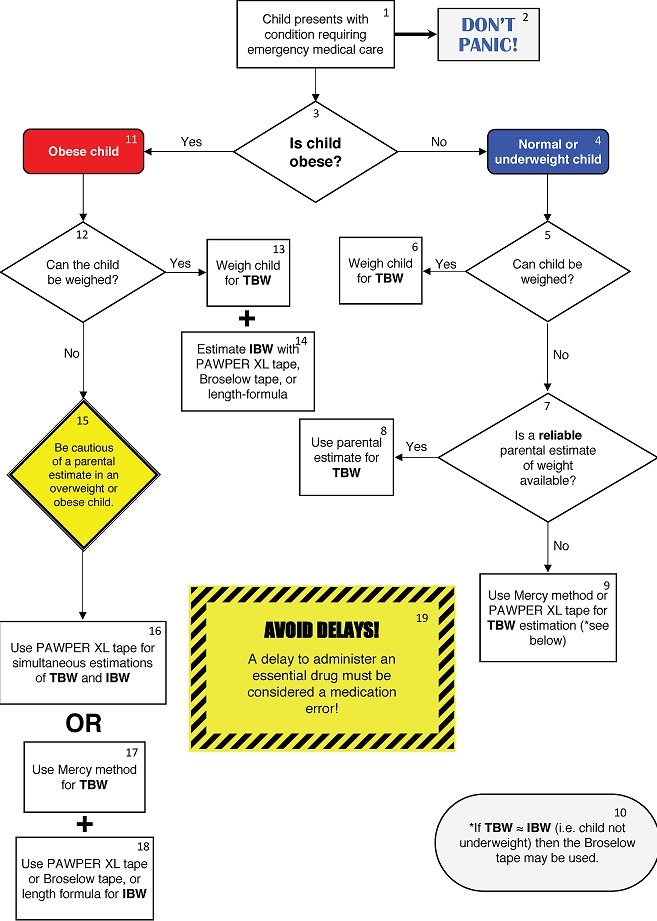
The full devised weight-estimation algorithm

**Figure 3:**
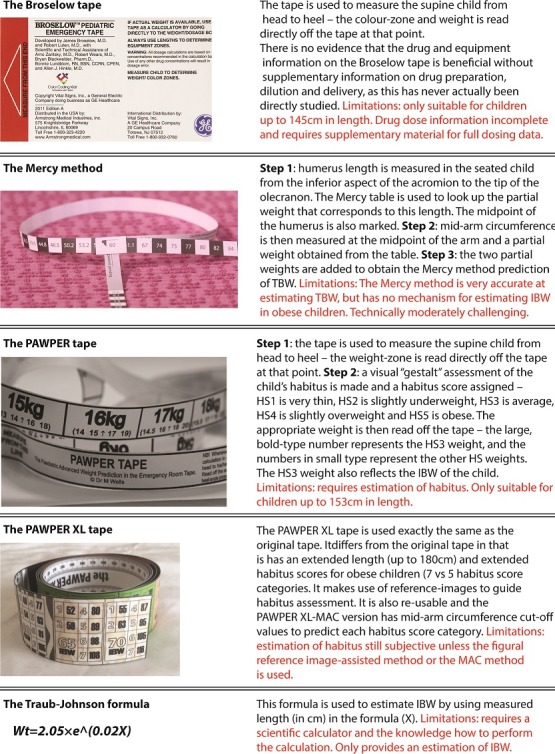
A description of the weight estimation systems included in the algorithm

The hierarchy of the preferred weight estimation methodologies is shown in the Figure 1. The devised algorithm is shown in Figure 2, with accompanying notes in Table 2. A description of the weight estimation systems included in the algorithm is contained in Figure 3.

**Table 2:** Notes on the weight estimation algorithm

	Comment
1	A critically ill or injured child in need of time-sensitive medical intervention.
2	Don’t panic! But call for help if it is required!
3	The weight estimation strategy will depend on whether the child is obese or not. This decision must initially be based on a visual inspection of the child to estimate their weight status. The use of a validated method of body habitus determination is preferred e.g. figural reference images, anthropometric measurements or the use of the PAWPER XL-MAC method with mid-arm circumference measurement.
4	If the child is not obese then follow this arm of the algorithm.
5	If the child does not require immediate emergency care and can safely be mobilised then the child should be weighed.
6	A calibrated scale may be used to obtain an accurate measurement of TBW in kg.
7	If the child cannot be weighed and the regular caregiver of the child is present, then a parental estimate of weight may be considered
8	A parental estimate of weight may be used if the regular caregiver of the child is present, they are not overwrought, if the child has recently been weighed by them or in their presence and they are confident that they can accurately remember the weight.
9	If a parental estimate of weight is not available, then one of the dual length- and habitus-based methods of weight estimation should be used. Both the Mercy method and the PAWPER XL tape have been shown to be highly accurate in normal weight and underweight children. These methods can provide a weight estimation in children up to 16 years of age.
10	If the child is not underweight (i.e. TBW is similar to IBW) then the Broselow tape may be used if the child is less than 145cm in length. If not, the Mercy method or PAWPER XL tape should be used.
11	If the child is obese (as determined by rapid visual inspection, the use of reference figural images, or the PAWPER-XL tape with mid-arm circumference method) then follow this arm of the algorithm.
12	If the child does not require immediate emergency care and can safely be mobilised then the child should be weighed.
13	A calibrated scale may be used to obtain an accurate measurement of TBW in kg.
14	A measured weight will only provide an accurate value for TBW. A method for estimating IBW will therefore still need to be used: the PAWPER XL tape, the Broselow tape or a length-based formula (such as the Traub-Johnson formula) may be used. Growth chart methods of estimating IBW may also be used if there is no medical emergency.
15	Parental estimates of weight have not been validated in obese children. Parents frequently underestimate weight and weight status in obese children – this method should be used with caution.
16	The PAWPER XL tape may be used to generate estimates of TBW and IBW immediately and simultaneously.
17	Alternatively, the Mercy method may be used to generate an estimate of TBW. The Mercy method may be more accurate than the PAWPER XL tape for estimating TBW in severely obese children.
18	The Mercy method cannot generate an estimate of IBW. The PAWPER XL tape, the Broselow tape (for children < 145cm in length) or a length-based formula (the Traub-Johnson formula) must be used in addition to the Mercy method.
19	During emergency medical care it is essential to avoid delays. Omitting an essential medication because of an inability to rapidly obtain an estimation of weight is a medical error.

## Discussion

This is the first article to suggest a methodology to guide the practice of weight estimation in obese children, which is more complex than in non-obese children. Even though medication errors in children have been shown to be common and serious, little work has been done to evaluate how inaccuracies in weight estimation contribute to these errors and how this could be improved, especially in obese children [2, 20].

### Appropriate dose scalars for obese children

Estimates of both TBW and IBW are required to permit safe and effective drug dosing in obese children. This has received little attention in emergency weight-estimation studies, but it is important as, notwithstanding the emergency nature of any presentation, it is imperative that medication errors be minimised. With the high prevalence of childhood obesity in high-, middle- and low-income countries, it is not uncommon that obese children will present with conditions that require urgent treatment. Errors in the selection of the dosing scalar for drug dose calculations could result in a significant dose error. The inappropriate use of TBW for hydrophilic drugs could cause significant overdosing and potential resultant harm. The inappropriate use of IBW for lipophilic medications would result in a significant underdosing, with potential diminished effectiveness of drug treatment and potential harm – e.g. the failure to rapidly abort seizures in status epilepticus with inadequate doses of valproate. It is important to recognise that IBW should not be used for non-obese children. Although IBW and TBW are similar for children around the 50th centile of weight-for-length, IBW should definitely not be used for thin or underweight children as IBW will exceed TBW and may result in a dangerous overdosing of medications [21].

**Figure 4:**
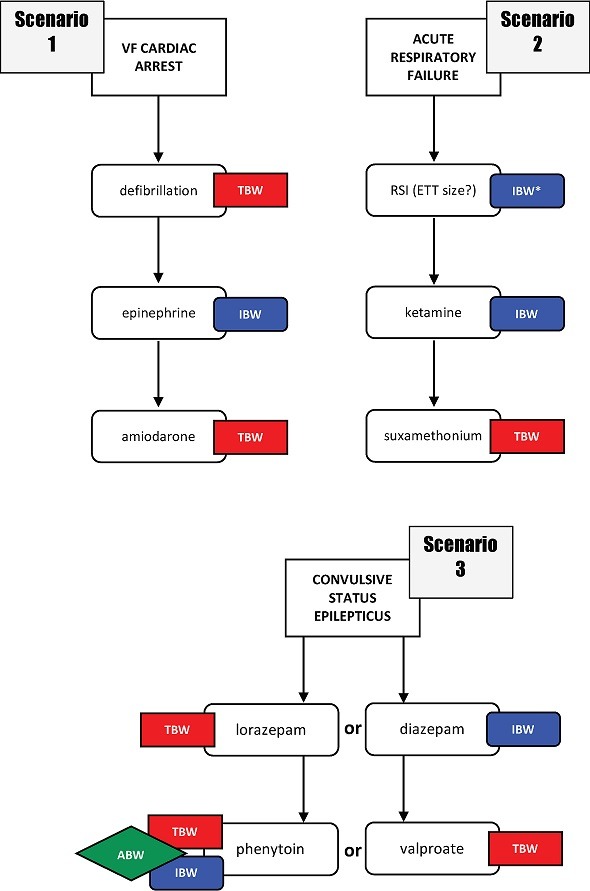
Three clinical scenarios highlighting the time-sensitive need for both TBW and IBW in an obese child

**Figure 4** demonstrates the different weight scalars required during emergency medical management of an obese child in three common emergency scenarios. These scenarios illustrate not only the importance of estimating both TBW and IBW, but also how early these estimates are required after the initiation of emergency care. The first scenario a child in cardiac arrest illustrates the need for TBW for determination of defibrillation doses and for doses of lipophilic medications (e.g. amiodarone). The correct management for a child presenting with ventricular fibrillation (VF) would be immediate defibrillation. A measure of TBW would therefore be required immediately to enable the calculation of the correct defibrillation energy dose. The initial dose of amiodarone would be required four to five minutes into the resuscitation. IBW is required to scale doses of hydrophilic medications (e.g. epinephrine) – dosing these medications to TBW carries a high risk of morbidity in obese children. Since the correct management of a child with a cardiac arrest would require the administration of epinephrine within two to four minutes of the initiation of the resuscitation, an accurate estimation of IBW would be required very early in the process to facilitate correct dose calculation.

The second scenario illustrates a similar requirement to have estimations of both TBW and IBW to allow for optimum drug dosing. While IBW is not directly useful for determining equipment size, length is. Therefore, the non-habitus-modified weight (IBW) is more useful to determine equipment size than a habitus-modified TBW estimation. In this scenario, where rapid airway management is required, drug doses would need to be determined immediately to allow for their preparation during the period of pre-oxygenation. There would be very little leeway for delay in obtaining estimates of both TBW and IBW to allow for optimum management.

The third scenario, an obese child with status epilepticus, illustrates further complexities in drug dose calculation. As in the other scenarios, both TBW and IBW estimations are required within the first few minutes of initiating emergency care – there is no time for delay. Anticonvulsant medications must be dosed according to TBW or IBW depending on their individual pharmacokinetic properties. When anti-epileptic medications are administered, doses also need to be individualised: some drugs, such as phenytoin, are best dosed to an adjusted body weight (ABW) for which estimations of both TBW and IBW are required. Other drugs, such as valproate, must be dosed to TBW.

### Weight estimation in obese children

The decision on which weight estimation methods to include in the algorithm depended primarily on their established accuracy. Their role within the framework of the algorithm was determined primarily by how they function and how easy they are to use. The dual length- and habitus-based systems are significantly more accurate than univariate age-based or length-based systems: despite being slightly less accurate in obese children than non-obese children, the Mercy method and the PAWPER XL tape remain the most accurate weight-estimation tools available. How these systems are used has been described elsewhere [22-25].

### The algorithm

The rationale behind the proposed algorithm was straightforward – to provide a method to quickly and accurately obtain estimates of an obese child’s TBW and IBW within the first few moments of initiating resuscitative care.

### Key elements of the algorithm

The identification of the obese child is at the crux of this algorithm, but it can be difficult even for experienced healthcare providers to identify obesity accurately [17]. Ideally, a validated system of assessing habitus should be employed such as reference images, or anthropometric measurements (e.g. mid-arm circumference or waist circumference) [26, 27]. This may add slightly to the time taken to follow the algorithm, but will increase the accuracy and appropriateness of management. The reliability of parental estimates has not been established in obese children and should be considered only if the parent can confidently recall a very recent measured weight. Parents cannot accurately assess the weight status of their children and may underestimate weight when asked to estimate. The user of this algorithm must weigh up the use of one method of weight estimation against the use of multiple methods. The Mercy method is slightly more accurate than the PAWPER XL tape in estimating TBW in severely obese children, but it cannot predict IBW. The clinical scenario may dictate which method or methods are most appropriate: the speed with which a weight-estimation is required (e.g. to calculate a defibrillation dose versus to calculate an induction dose for rapid sequence intubation) may also impact this decision. It would be beneficial if clinicians were proficient with more than one technique.

### Prerequisites for success: overcoming complexity

Converting a quantification of body weight into the correct volume of diluted drug to administer is a complex, cognitively-intense process. It cannot succeed reliably without the use of comprehensive drug-dosing resuscitation aids, which provide information on drug dilution, preparation and required volume-to-administer. In obese children, this is far more complex, because of the need to use the correct weight descriptor for each drug (TBW or IBW). This would preferably be achieved using a computerised system or mobile phone application to limit the potential for error. Further research is still required in this field to determine the feasibility of such a system for use in both obese and non-obese children.

### Obstacles to emergency weight estimation

There are two main obstacles to optimum drug therapy in emergencies, which are a reflection of a statement by the Institute of Medicine in the USA: “Meet the enemy: he is us” [28]. The first obstacle is scepticism, the second is a lack of professionalism.

### Scepticism

There is an argument that the lack of high-grade evidence for many paediatric drug dose ranges means that it is pointless to attempt to estimate weight accurately. It is true that not enough is known about drug doses in children, but the clinician must nonetheless attempt to reduce errors as much as possible [7]. To use a system of weight estimation that is known to be inaccurate must be considered poor medical practice [29]. It is therefore essential that the most accurate and appropriate weight estimation systems be used.

### Professionalism

A lowered standard of care should not be tolerated during emergencies. Even the most basic weight estimation techniques have been shown to be vulnerable to error without appropriate instruction and practice, so training in weight estimation should become routine [30]. Prompt defibrillation is emphasised, taught and trained for the treatment of arrythmogenic cardiac arrest, but the preceding, paralysing, fumbling delay in weight estimation to allow for energy dose calculation is frequently overlooked in the teaching. Weight estimation and drug dosing needs to be integral to the teaching and training for resuscitation. In this way weight estimation will not be an impediment to medical care, but will enhance it. This could happen in two ways. Weight estimation procedures should form part of simulation training drills so that healthcare providers become familiar with how to integrate them into acute medical care and how best to use resuscitation aids. Weight estimation procedures could also be performed on children with less acute presentations, together with feedback from actual measured weight, so that healthcare providers can become more experienced, and more accurate, with the use of their preferred systems.

### Limitations

Given the lack of evidence on this topic, it was impossible to provide more than low-grade recommendations on how weight estimation could be used in a resuscitation setting.

## Conclusion

A greater emphasis should be placed on the role of weight estimation in paediatric resuscitation: an integrated, comprehensive weight-estimation-resuscitation aid system can facilitate emergency medical care and reduce cognitive stress and medication errors. A poorly-planned weight estimation and drug dosing strategy can inject impediments into medical care with a high consequent risk of medical errors. Training in every element of resuscitative management, including weight estimation, is essential to deliver the level of professional care that our patients deserve. Differences in obese children’s body composition should be respected and dealt with according to pre-planned methodology, making use of appropriate methodologies to estimate or measure TBW and IBW when indicated. This algorithm is a preliminary proposition, designed to assist the training and preparation of teams whose task it is to resuscitate children. This will need to be critically examined, tested in simulation and improved upon. Most importantly, this proposal is intended to draw attention to the need for professionalism in emergency care: to advocate for the appropriate use of weight estimation and to promote training in weight estimation procedures to ultimately reduce medical errors in paediatric resuscitations.

### What is known about this topic

- The three most accurate weight estimation systems at present are estimates by parents, the Mercy method and the PAWPER tape system;
- Weight estimation can be a barrier to the provision of successful emergency care;
- Training is essential to enable healthcare providers to use weight estimation systems effectively.

### What this study adds

- This study provides a unique method of training in weight estimation for emergency care;
- This study also provides a first-of-its-kind algorithm to allow the user to select the most appropriate method of estimating weight in children of different habitus;
- This study also provides evidence-based guidelines on the use of total and ideal body weight for drug dose calculations.
